# Clinical outcomes and timing on the combination of focal radiation therapy and immunotherapy for the treatment of brain metastases

**DOI:** 10.3389/fimmu.2023.1236398

**Published:** 2023-10-17

**Authors:** Gabriela Antelo, Silvia Comas, Francesc Casas, Izaskun Valduvieco, Tanny Barreto, María Laplana, Joel Mases, Gabriela Oses, Meritxell Mollà

**Affiliations:** ^1^ Radiation Oncology Department, Hospital Clinic Barcelona, Barcelona, Spain; ^2^ Radiation Oncology Department, Institut Catalá d'Oncologia (ICO), Badalona, Badalona, Spain

**Keywords:** brain metastases, stereotactic radiation, radiosurgery, immunotherapy, combination (combined) therapy

## Abstract

**Introduction:**

Radiotherapy is one of the standard treatments for brain metastases (BM). Over the past years, the introduction of immunotherapy as routine treatment for solid tumors has forced investigators to review and evaluate how it would interact with radiation. Radiation and Immunotherapy have shown a synergic effect activating the host’s immune system and enhancing treatment response. The combinatory effect on BM is currently under investigation.

**Methods:**

Data published on Pubmed to determine toxicity, survival, treatment characteristics and timing on the combination of radiotherapy and immunotherapy for the treatment of BM has been reviewed.

**Results:**

Mostly retrospective reviews report an improvement of intracranial progression free survival (iPFS) when combining radioimmunotherapy for BM patients. Two systematic reviews and meta-analysis and one phase II prospective trial also report a benefit on iPFS without an increase of toxicity. Among the published literature, the definition of concurrency is heterogeneous, being one month or even narrowed intervals correlated to better clinical outcomes. Toxicity due to concurrent radioimmunotherapy, specifically symptomatic radionecrosis, is also directly analyzed and reported to be low, similar to the toxicity rates secondary to stereotactic radiosurgery alone.

**Conclusion:**

Radiation combined with immunotherapy has shown in predominantly retrospective reviews a synergic effect on the treatment of BM. The concurrent combination of radioimmunotherapy is a feasible therapeutic strategy and seems to improve clinical outcomes, especially iPFS, when delivered within <30 days. Larger prospective and randomized studies are needed to establish reliable outcomes, best delivery strategies and toxicity profile.

## Introduction

Brain metastases (BM) are the most frequent brain tumor, accounting for a high morbidity and mortality among patients. It is estimated that around 20-40% of oncologic patients will develop brain metastases at some point of their disease. The most common origins are small and non-small cell lung cancer (NSCLC), breast and melanoma cancer (1). For the past decade, multiple advances in systemic treatments, availability of more sensitive radiological imaging and the remarkable technical improvement for delivering radiation treatments, have increased oncologic patients’ survival, and therefore, the incidence of brain metastases has increased as well.

Historically, surgery and radiotherapy (RT) have been the mainstay treatments for brain metastases, while systemic treatments were set aside given their limited ability for crossing the brain blood barrier (BBB). Immune checkpoint inhibitors (ICIs) have emerged as a promising option due to their improved ability to cross BBB. Nevertheless, its effect on intracranial metastases is lower compared to extracranial disease (2). Therefore, local treatments remain as the cornerstone of the therapeutic approach for BM.

Combining ICI with focal stereotactic radiotherapy techniques has resulted in improved outcomes on patients with different extracranial primary tumors and more recently, research efforts have been made to establish its efficacy on primary and secondary brain malignancies.

A methodical review of the literature was executed searching on bibliographic online database site, Pubmed. The keywords used, separately and in combinations, were: “immunotherapy”, “stereotactic radiotherapy”, “radiosurgery”, “fractionated stereotactic radiotherapy”, “combination”, “concurrent”, “brain metastases”.

## Biological effect of radiation and it’s combination with Immune checkpoint inhibitors

Pre-clinical and clinical evidence has demonstrated that RT performs its tumoricidal action through three different mechanisms that occur synchronously. It is well known that radiation induces cytotoxic tumor cell death through DNA strand damage leading to cell cycle arrest and nowadays we are aware of an independent bystander effect resulting from interactions with the tumor microenvironment and host’s immune system. This effect seems to be stronger when higher ionizing radiation doses are delivered through high-precision techniques (3). Immunological effects of radiation in extracranial malignancies have been well established.

Radiation causes immunogenic cell death and a consequent release of tumor related antigens associated with stress and cellular damage (4–8). The release of these antigens activates antigen recognition by dendritic cells (DCs) which will migrate to lymph nodes, leading to a subsequent tumor-specific T cell activation and proliferation. Furthermore, RT leads to recruitment of immune cells to tumor microenvironment and enhanced cross-priming of DC (9–12).

Conversely, RT has a concomitant potential inhibitory effect on immune system which include up-regulation of programmed cell death ligand (PD-L1) among other mechanisms (13–15). This effect represents a good opportunity to consider adding an ICI looking for synergic action.

In the brain scenario, the cellular and molecular interactions between radiation and the brain are still partially unknown. The inflammatory tumor microenvironment of BM differs from extracranial metastasis due to a series of unique structural and functional characteristics and the fact that immune responses are tightly regulated. We must consider the BBB, which under normal circumstances constitutes an efficient containment system that prevents free penetration of immune cells into the brain. Antigen presentation in the brain is challenged due to the constitutive absence of expression of the MHC-1 molecules in the neurons of the adult brain and the presence of a limited number of antigen-presenting cells (16). It has been described that microglia are the main component involved in the innate immune response and show multiple similarities with DC and macrophages, being able of antigen presentation. Microglia present receptors that are also able to sense danger signals from their environment (17, 18). Conventional DC are found too in specific regions of the brain (19).

In normal conditions, neurons generate factors that keep microglia inhibited. Radiation leads to microglia activation by stimulating the secretion of pro-inflammatory cytokines and expression of stress signals on neurons’ surface. Additionally, radiation disrupts BBB, leading to increased permeability, especially when delivering high focal radiation doses. Activated microglia will secrete CCL2, which is a chemoattractant for CCR2-expressing peripheral macrophages that will penetrate the BBB due to the acquired increased permeability (20). All these signals activate brain-residing DC which will migrate through the lymphatic drainage of the cerebrum spinal fluid (CSF) and to regional lymphatic ganglions, where they will interact and activate T cells which subsequently will penetrate the BBB (21).

Tumor cells are endowed with mechanisms that allow them to evade the host’s immune system, including checkpoint molecules. Programmed cell death protein 1 (PD-1) and its ligand (PD-L1) as well as T-lymphocyte-associated protein 4 (CTLA-4) have been the most evaluated and approved ICI. Microglia and macrophages have also shown to express immunosuppressive factors like PD-L1 (22). Nivolumab, an anti-PD1, and ipilimumab, anti-CTLA-4, has shown potential to penetrate the CSF by binding the peripherally activated T cells crossing the BBB (23).

In summary, RT leads to enhanced neoantigen release, promotion of inflammatory signals, improvement of cross-antigen presentation, increased T cell tumor infiltration and generation of specific T-cell response. Additionally, radiation to the brain disrupts BBB, leading to increased permeability, especially when delivering high focal radiation doses (24, 25). Combination of RT and ICI have a synergic effect activating host’s immune system, with potential to subvert immune evasion and enhance treatment response.

## Clinical outcomes of radioimmunotherapy for the treatment of brain metastases

Over the past decade, the positive results of immunotherapy for some extracranial pathologies have led to great interest in the study of its efficacy for intracranial pathologies, especially for the treatment of BM in combination with high-dose focal RT. Literature concerning radioimmunotherapy for the treatment of BM is mainly based in retrospective reviews and meta-analyses, mostly including melanoma and NSCLC patients.

A meta-analysis of 19 studies evaluating the efficacy of the combination, reported better systemic efficacy with concurrent treatment compared to RT alone or sequential RT + ICI. Overall survival (OS) and intracranial progression free survival (iPFS) tend to improve with the combination, but statistically significant differences in OS were only found for NSCLC patients, also favoring concurrent treatment (26). Another meta-analysis of 8 retrospective studies reported that concurrent stereotactic radiosurgery (SRS) + ICIs conferred a significant 12-month OS benefit compared to no concurrent strategy in patients treated for melanoma BM (odds ratio (OR) = 1.74; p = 0.011) with comparable 12-month local progression free survival (LPFS) (OR = 2.09; p = 0.154) and iPFS (OR = 0.88; p = 0.839) (27). The meta-analysis of Lehrer et al., also supported concurrent combination of ICI+SRS (<30 days) as the best option for achieving an improved OS, local and regional brain control at one year (28).

Porte et al. analyzed 51 patients with 84 BM from NSCLC. One-year iPFS did not reach statistical significance but showed a tendency to be superior when delivering both treatments concurrently (within a month), compared to SRT delivered before or after ICI (49.6% vs 24.1% and 34.2%, respectively, p=0.09). No differences in Local control or OS were found between groups. Le et al. reported improvement in iPFS for 144 patients with **NSCLC** and melanoma BM treated with SRS within 30 days of ICI administration (29). The publication of Koening confirmed improvement in one-year OS with concurrent treatment (<4 weeks) compared to non-concurrent (48.6% vs 25.4% respectively, p= 0.044). Extracranial failure at 1 year also supported the concurrent strategy (69.7% vs. 80.8%, p= 0.007) (30).

Some groups have addressed BM volume response to combinatory treatment with SRS+ICI. Qian et al. retrospectively reviewed 33 melanoma BM treated with SRS (range 12-24 Gy) within 4 weeks from ICI administration. The concurrent combination showed a greater median percent reduction in lesion volume at 1.5 months (-63.1% vs -43.2%, p<0.0001), at 3 months (-83.0% vs -52.8%, p<0.0001), and at 6 months (-94.9% vs -66.2%, p<0.0001) compared to non-concurrent treatment patients from the same review (31).

More recently, a phase II trial analyzed the efficacy and safety of SRS combined with Nivolumab for BM from NSCLC and renal cell carcinoma (32). Twenty-three patients, with a median of 3 BM (1–9) per patient, received SRS to every BM within 14 days from Nivolumab first dose administration. One-year iPFS and OS were 45.2% (95% CI 29.3–69.6%) and 61.3% (95% CI 45.1–83.3%) respectively, showing an improvement on iPFS comparing to historical controls treated with SRS alone, which reported taxes of 16-27% (33, 34). Even when including patients with >3 BM, the combination showed one year intracranial control rates comparable to those reported by Brown adding WBRT to SRS in patients with 1-3 BM. Intracranial PFS improved also compared to Nivolumab monotherapy, with reported 6 months iPFS of 23.8% (95% CI, 11.1% to 39.2%) (35) and to Pembrolizumab monotherapy with reported one year iPFS of 33% (95% CI 19-56%) (36).

Recent published evidence is summarized on **Table 1**.

**Table 1:** Summary of recent published evidence.

Author	Year	Type	N	Nº Met	Primary tumor	CC definition	Local control	Distant brain control	OS	Immunotherapy	Radiation dose Median
Qian	2016	Retro	79	566	Melanoma	<4 weeks	Volume reduction (1.5-3-6m) CC→63.1-83-94.9% nonCC→ 43.2-52.8-66.2%	N/S	CC: 19.1 m non-CC: 8.0 m P= 0.08	Anti CTLA4 (72%) Anti PD 1 (28%)	SRS 20 Gy
Skrepnik	2017	Retro	25	58	Melanoma	<30 days	N/S	1y CC->75% nonCC-> 23.5% P= 0.03	N/S	Ipilimumab (100%)	SRS 21 Gy
Koening	2019	Retro	97	580	Mixed	<4 weeks	CC 4% nonCC 3%	CC 66% nonCC 72%	1y CC-> 49% nCC-> 25% P= 0.05	Nivolumab (43.1%) Pembrolizumab (22.9%) Ipilimumab (13.3%) Other (20.7%)	SRS 22 Gy (91.6% fSRT: 3f- 27 Gy (7.1%)
Kotecha	2019	Retro	150	1003	Mixed	Immediate: <1 half-life concurrent: <5 half-lives	BOR: P= <0.001 - Immediate: 100% - Concurrent: 57% LC 12m: - CC: 86% - nonCC: 65%	Immediate: 71% Non-Immediate: 53% P= 0.008 CC: 59% non CC: 56% P= 0.34	12m OS CR: Not Reached. PR: 27m SD: 25m P= 0.004	NIvolumab (79%) Pembrolizumab (15%) Atezolizumab (7%) Avelumab (1%)	N/S
Murphy	2019	Retro	26	90	Melanoma	<30 days	2y: 95.4% No differences bt groups	CC: 19m nonCC: 3.4m P= <0.001	median: 26.1m (entire cohort)	Ipilimumab (N/S%) Nivolumab (N/S%) Pembrolizumab (N/S%)	SRS 22 Gy fSRT 27-30 Gy in 3-5fx
Lehrer	2022	Retro	203	1388	Melanoma	<4 weeks	1y LC: - CC 81.9% - nonCC: 74.6% P= 0.36	N/S	Median OS: CC: 36.1 m nonCC: 19.8 m P= 0.051	Ipilimumab (N/S%) Nivolumab (N/S%) Pembrolizumab (N/S%) Other & combinations (N/S%)	SRS 20 Gy
Le	2022	Retro	144	477	NSCLC (66%) Melanoma (34%)	<30 days	N/S	HR 0.15	N/S	Ipilimumab (34,2%) Nivolumab (39.5%) Pembrolizumab (13.2%)	Median 22 Gy
Porte	2022	Retro	51	84	NSCLC	<1 month	78.9% concurrent 70.% before 77.8% after	48% concurrent 24.1% before 22.8% after P= 0.031	Median 18m	Nivolumab (47.1%) Pembrolizumab (33.3%) Durvalumab (15.7%) Atezolizumab (3.9%)	SRS 15-18 Gy fSRT 18-27 Gy/3-5fx
Wong	2023	Prospective Phase II	26	Median 3 m/pt	NSCLC (85%) RCC (15%)	<14 days	70%	1 year Accumulated risk : 19.5%	Median 21.4m	Nivolumab (100%)	15-21 Gy

Summary of publication on efficacy. Retro, retrospective review; N/S, non specified; CC, concurrent treatment; nonCC, non concurrent treatment; SRS, stereotactic Radiosurgery; fSRT, fractionated Stereotactic Radiotherapy; fx, fraction; WBRT, Whole brain radiotherapy; NSCLC, non-small cell lung cancer; RCC, Renal cell carcinoma; BOR, best objective response. CR, complete response; PR, partial response; SD, stable disease; m/pt, metastases per patient.

### Timing for combining radiotherapy and immune checkpoint inhibitor

It is unclear how to select the optimal strategy for combining RT and ICI to achieve the highest synergic effect while minimizing toxicity of both treatments. Radiation alone produces a delayed effect which eventually modifies tumor microenvironment as well as modifies the permeability of the BBB, facilitating the entrance of systemic treatment into the central nervous system. This time-dependent effects of radiation leads to the hypothesis that different clinical outcomes and toxicity could be obtained depending on when radiation and immunotherapy are delivered. Knowledge on physiology suggests a time dependent interaction that has been confirmed in published literature. Regarding time lapse between SRT and ICI, the definition of concurrent strategies in literature is quite heterogeneous. The period most frequently used to define concurrency was 1 month or 30 days.

Le et al. at. classified patients in their review following a variety of definition in the literature based on the time lapse between SRS and ICI administration into 3 separated groups: those treated with SRS within 30 days from ICI, those treated with SRS between within 31-60 days from ICI and those who received SRS within 60-90 days from ICI. On multivariate analysis the narrowed period (<30 days from SRS to ICI) was associated with improved control on distant brain failure, therefore less than 30 days was the time period selected to perform the complete analysis and discussion (37).

Kotecha et al, defined an “immediate group” when SRS was given within <1 half-live of the ICI, and a “concurrent group” when it was delivered within <5 half-lives of ICI. Complete response rate (50% vs 32%, p=0.042) and 12-month durable response (94 vs 71% p<0.001) improved with “immediate” strategy. When comparing any of these concurrent groups with sequential treatment, concurrent strategy showed a better response at 12 months (86% vs 65%) (38).

Chen et al, defined an even narrowed period of concurrency that consisted in SRS given 2 weeks within ICI. NSCLC, renal cancer and melanoma BM were included in the review. Multivariate analysis reported superior OS when concurrent treatment was prescribed compared to non-concurrent (39).

### Toxicity in combination immune checkpoint inhibitor and radiation therapy for brain metastases

A relevant consideration for oncologists to indicate the combination of RT and immunotherapy for the treatment of BM is the potential risk of toxicity. Evaluation of the toxicity should consider immunotherapy-related adverse effects, radiation-related adverse effects and potential adverse effects derived from the interaction of both treatments. It is also important to analyze the toxicity profile according to the timing on which different treatments are delivered.

Koenig et al. defined radiation necrosis (RN) as treatment-related imaging changes in the presence or absence of symptoms. For 97 patients, RN occurred in 32% of resection cavities and 4% of intact lesions (P < 0.001). Concurrency within 4 weeks was associated with higher rate of RN at one year (6.4% vs. 2.0%; p= 0.005) (30). However, this RN rates were comparable to previously published rates with SRS alone (40, 41).

Whether most of published literature included non-symptomatic imaging changes suspicious of RN, Weingarten et al. considered that the advent of symptomatic RN was especially clinically relevant in those patients receiving Radioimmunotherapy. Corticosteroids, the most common treatment for symptomatic RN, may interfere with the ICI mechanism of action and reduce the immunostimulatory effect, resulting in reduced effectiveness of the treatment. Weingarten et al. considered symptomatic RN when patients presented with characteristic RN imaging findings in addition to clinical symptoms and showed improvement of symptoms when receiving corticosteroids, bevacizumab, or surgical resection (with pathology confirming RN) and no progression of the lesion was observed. This group retrospectively reviewed 57 patients with a total of 387 irradiated BM. They reported a rate of symptomatic RN of 7%. The rate of symptomatic RN per lesion treated when combining both treatments was 1.6% with a median time to develop it of 150 days (42).

In the study of Cabanie et al. the only significant predictive factor of RN was tumor volume (OR = 0.94, 95% CI = 0.87–1.00, P = 0.032). This group evaluated 103 BM treated with an anti-PD1 or anti-PDL1 and/or anti-CTLA4 in the month before and/or after cerebral SRT. Reported rate of neurological grade ≥ 3 toxicities was 5.1% (intracranial hypertension) and 9.7% for RN (with 20% being symptomatic cases) (43).

Combining SRS and Nivolumab has been described as safe in Wong’s phase II study (32). Among 23 cases, they observed two grade 3 fatigues and two patients needed onset or an increased dose of dexamethasone to control headache and local brain edema. A 95% CI of 0–13% for developing RN has been reported and no significant neurocognitive deficit differences were found from baseline to 3 and 6 months after combined treatment.

To date, we do not have a well-defined toxicity profile for the RT-ICI combination for the treatment of BM and no clinical consistently predictive factors have been established. Published literature suggests that their combination is safe, with the risk of RN being the most reported adverse effect, although apparently similar to the rate obtained with RT in monotherapy. Further investigation and phase III trials derived evidence is needed.

The neurocognitive effect of combining stereotactic focal irradiation with immunotherapy is, to date, poorly valued in the literature due to the lack of information from standardized neurocognitive and quality of life tests performed on patients. To address this issue, it is necessary to have the results of randomized studies with a standardized neurocognitive assessment of all participants.

Toxicity described in the literature is summarized on **Table 2**.

**Table 2:** Summary of toxicity published .

Author	Year	Type	Nº Patients	Nº Metastases	Primary tumor	RT dose	Previous Brain treatment	Immunotherapy	Symptomatic Radionecrosis	Other Toxicities
Weingarten	2019	Retro	57	387	Mixed	Median 20 Gy	WBRT (5%)	Nivolumab (58%) Ipilimumab (18%) Pembrolizumab (21%) Durvalumab (2%) Other (4%)	7%	N/S
Koening	2019	Retro	97	580	Mixed	SRS 22 Gy (91.6% fSRT: 3f- 27 Gy (7.1%)	N/S	Nivolumab (43.1%) Pembrolizumab (22.9%) Ipilimumab (13.3%) Other (20.7%)	CC: 8% nonCC: 2% P=: 0.001	N/S
Cabaine	2021	Retro	59	103	NSCLC (60%) Melanoma (40%)	SRS: 20 Gy fSRT: 33 Gy 3fx	N/S	Nivolumab (61.2%) Pembrolizumab (30.1%) Other (8.7%)	CC (<7 days): 0% CC(8-14 days): 13.1% CC(>15d):8.3%)	Intracranial hypertension (5.1%).
Le	2022	Retro	144	477	NSCLC (66%) Melanoma (34%)	Median 22 Gy	WBRT (29%) Surgery (18.1%)	Ipilimumab (34,2%) Nivolumab (39.5%) Pembrolizumab (13.2%)	3.8%	Hemorrhage 1.9%
Porte	2022	Retro	51	84	NSCLC	SRS 15-18 Gy fSRT 18-27 Gy/3-5fx	No	Nivolumab (47.1%) Pembrolizumab (33.3%) Durvalumab (15.7%) Atezolizumab (3.9%)	No differences bt CC and nonCC	N/S
Lehrer	2022	Retro	203	1388	Melanoma	SRS 20 Gy	No	Ipilimumab (N/S%) Nivolumab (N/S%) Pembrolizumab (N/S%) Other & combinations (N/S%)	CC: 9.4% nonCC: 8.2% P= 0.766	N/S
Wong	2023	Prospective Phase II	26	Median: 3 m/pt	NSCLC (85%) RCC (15%)	15-21 Gy	No	Nivolumab (100%)	0%	G3 Fatigue (7.7%)

Summary of publication on toxicity. Retro, retrospective review; N/S, non specified; CC, concurrent treatment; nonCC, non concurrent treatment; SRS, stereotactic Radiosurgery; fSRT, fractionated Stereotactic Radiotherapy; fx, fraction; WBRT, Whole brain radiotherapy; NSCLC, non-small cell lung cancer; RCC, Renal cell carcinoma; m/pt, metastases per patient.

## Conclusions

Stereotactic radiosurgery (SRS) or focal hypofractionated radiotherapy (FHFRT) are standard options for the management of BM, alone or in combination with surgery (44–46). For patients with more than 4 lesions whole brain radiotherapy (WBRT) is still considered the standard of care even at the risk of neurocognitive decline. Current indication of SRT for the treatment of patients with few BM has led to a slightly lower regional PFS compared to that achieved with classical WBRT, but with no effect on OS (3). Treatment modality recommendations have been mostly based on disease prognosis as well as number, size and location of BMs. Immune checkpoint inhibitors (ICIs) have emerged as a promising option due to their ability to cross brain blood barrier (BBB) and have become the standard of care for multiple metastatic cancers. Its combination with STR is believed to develop a synergic effect, beneficial for tumor control and diminishing the risk of regional recurrence.

Interpretation of the current published literature presents certain limitations due to the retrospective nature of most part of the studies. According to literature the concurrent combination of SRT and ICI seems to lead to better iPFS compared to sequential treatments or SRT alone, with some studies reporting benefit on OS. Current evidence supports this combination, although we still lack high-level evidence due to the absence of large prospective randomized phase III trials.

Published evidence suggests better clinical results when using times between treatments lower than 30-day period. Nevertheless, the diversity regarding the time-period to define concurrency across different studies highlights the need for standardized definitions and criteria to guide concurrent radiation and ICI delivery in clinical practice and research.

Even though combined RT and ICI for BM has been reported to be safe and well tolerated, the evidence from which it is derived is very low, and to date we do not have a reliable and well-established safety profile. One of the adverse effects that has aroused most interest is the development of symptomatic RN, especially due to the therapeutic management implications that it may entail and that could potentially negatively affect the efficacy of immunotherapy. In any case, we must not fail to consider the possibility of adverse events derived from the increase in ICI penetration itself at brain level.

We are at the gates of a change in the standard of care (SoC) of patients affected by BM. To date, research has shown that he combination of focal radiation therapy with immunotherapy is a safe and effective therapeutic strategy. Our patients will benefit from both SoC treatments without the need to sideline or forfeit the advantage of either focal radiation or immunotherapy.

However, stronger evidence and further research is needed with prospective trials with a larger number of patients to really confirm what we can only suggest today.

## Author contributions

GA and SC contributed equally to this work. All authors contributed to the article and approved the submitted version.
